# Experimental analysis of the humidification of air in bubble columns for thermal water treatment systems^☆^

**DOI:** 10.1016/j.expthermflusci.2020.110063

**Published:** 2020-02-13

**Authors:** Elias Eder, Markus Preißinger

**Affiliations:** illwerke vkw Endowed Professorship for Energy Efficiency, Research Centre Energy, Vorarlberg University of Applied Sciences, Dornbirn 6850, Austria

**Keywords:** Bubble column humidifier, Air humidification, Humidification-dehumidification, Desalination, Water treatment

## Abstract

The humidification-dehumidification process (HDH) for desalination is a promising technology to address water scarcity issues in rural regions. However, a low humidifier efficiency is a weakness of the process. Bubble column humidifiers (BCH) are promising for HDH, as they provide enhanced heat and mass transfer and have low maintenance requirements. Previous studies of HDH-systems with BCHs draw different conclusions regarding the impact of superficial air velocity and liquid height on the humidification. Furthermore, the impact of flow characteristics has never been investigated systematically at all. In this study, an optimized BCH test setup that allows for optical analysis of the humidifier is used and evaluated. Our test setup is validated, since the influence of water temperature on the humidification, which is exponential, is reproduced. Measurements with seawater show that the normalised system productivity is increased by about 56% with an increase in superficial air velocity from 0.5 cm/s to 5 cm/s. Furthermore, the system productivity is increased by around 29% with an increase in liquid height from 60 mm to 378 mm. While the impact of superficial air velocity can be traced back to temperature changes at the humidifier and dehumidifier outlets, the impact of liquid height is shown to be caused by a smaller heat loss surface in the humidifier with an increase in liquid height. For the impact of sieve plate orifice diameter, a clear influence on the humidification is not apparent, this parameter needs to be investigated further. Finally, our new test setup allows for analysing the humidification of air (1) in a systematic way, (2) in relevant measurement ranges and (3) in comparison with optical analyses of the flow characteristics.

## 1 Introduction

Desalination of seawater and brackish water is the only chance to provide the growing fresh water demand that we are facing on a global scale [1,2]. In rural and well-developed areas close to the sea, large-scale desalination units are combined with fossil fuel powered plants. Three different technologies are mainly applied for such areas: multistage flash distillation and multi-effect distillation as thermal technologies, reverse osmosis as electrical driven technology [3].

As these technologies are not economical on a small scale, alternative water treatment systems are needed for rural areas [4] Such systems need to fulfill three requirements [5,6]: (1) they are able to use solar energy, the most abundant form of energy in these areas, (2) they need low investment and maintenance costs, to be affordable on a low scale and (3) they are flexible to different kinds of feedstock: seawater, brackish water or even polluted ground or surface water.

The humidification dehumidification process (HDH) is a promising approach for small-scale desalination, as it satisfies these three re-quirements [7–10]. This distillation process is based on the natural water cycle: air is humidified through diffusion in direct contact with the untreated water in the humidifier; the humid air is subsequently cooled in the dehumidifier, and fresh water is collected as condensate.

One weakness of the HDH-process is a low humidifier efficiency [11]. In order to increase this efficiency, different humidifier designs, such as packed bed humidifiers, spray towers and wetted wall towers have been tested extensively in the past[12]. In recent studies, a new humidifier design has been proven to be a viable solution for HDH: the bubble column humidifier (BCH) [10,12,13].

The working principle of a BCH is straight-forward: air bubbles are dispersed into a liquid column, resulting in a high contact area between gaseous and liquid phase. BCHs have already been tested and evaluated regarding their applicability for fuel cells [14–16]. Their disadvantages, when applied for fuel cells, are (1) hard control of temperature and water vapor content of the outlet air, (2) moderate size, (3)condensation of water vapor at the humidifier outlet and (4) high effort for laboratory scale testing. However, most of these disadvantages are not relevant for water treatment purposes, since the goal in water treatment is to reach the maximum possible water vapor content at the humidifier outlet. Condensation of water vapor at the humidifier outlet is problematic, but can be overcome by heating the humidifier outlet air [16]. Generally, BCHs are a valid solution in water treatment as they provide an increased heat and mass transfer and less fouling in comparison with humidifier designs such as packed bed humidifiers [13,17]. However, a BCH can only make use of its inherent advantages, if geometric and operational parameters are chosen adequately. It is generally known, that crucial parameters are: (1) water temperature, (2) superficial air velocity, (3) liquid height and (4) flow characteristics, such as bubble size or gas holdup.

The dependence of the HDH-productivity on water temperature is set by thermodynamics: according to the Antoine equation, saturation pressure of air increases exponentially with an increase in air temperature, thus the productivity of an HDH-system follows this trend. In previous experimental studies, water temperatures in ranges between 30 °C and 87 °C have been investigated.

For the influence of superficial air velocity, which corresponds to the air mass flow rate, experimental studies contradict each other. On the one hand, most studies showed an enhanced humidification efficiency, respectively HDH-productivity, with an increase in superficial air velocity [8,17–23]. On the other hand, for experiments conducted by Liu and Sharqawy [24], a decrease in the humidifier efficiency is measured with increasing superficial air velocity. Furthermore, Behnam and Shafii [25] noticed a negative impact of the superficial air velocity on system productivity for the morning hours, yet a positive impact in the afternoon. This impact is reasonable, as it needs to be taken into account that the heat for their system was provided by evacuated tube collectors with heat pipes and was therefore dependent on the solar irradiation.

For the influence of liquid height, studies differ even further: while some studies determined an increase in humidification, if liquid height was increased [17,18], other studies stated, that the liquid height has a minor or even no influence on the humidification at all [8,24,21,23]. In the experiments of Abd-ur-Rehman and Al-Sulaiman [19], increasing the liquid height even led to a decreasing humidification in the bubble column. Measurements conducted by Srithar and Rajaseenivasan [20] also showed a decrease in system productivity caused by an increase in liquid height. Results by Behnam and Shafii [25] are of great interest, as they show an increase in productivity with liquid height, until the heat pipes providing the heat for the bubble column are fully submerged. A further increase in liquid height leads to a decrease in productivity, which indicates, that an increase in liquid height is not enhancing the humidification.

Table 1 gives information on the measurement ranges and the impact of the parameters, that have been investigated in the above cited experimental studies. The flow rate of the air and the liquid height have been varied in ranges that mostly do not overlap. This might be a reason for the contradictory results regarding the impact of these parameters on the humidification.

In summary, the existing literature on BCHs reveals three short-comings:

- **Insufficient investigation of flow characteristics**: despite indications in literature, that flow characteristics have a significant impact on the humidification in bubble columns [9,24], most studies did not look into this aspect. Only Khalil et al. [17] investigated flow characteristics to a certain extent by means of a variation of sieve plate orifice diameter.
- **Inadequate measurement ranges:** for an accurate characterisation of superficial air velocity and liquid height, the measurement ranges need to be chosen carefully. To estimate the impact of superficial air velocity, the transition from a laminar to a turbulent flow regime needs to be within the measurement range. As the liquid height variation by Liu and Sharqawy [24] was only 20 mm, it might not have been possible to notice an influence of liquid height on the humidification, in contrast to variations of 53 mm [18] and 150 mm [17].
- **Insufficient cross-verification and evaluation of data and boundary conditions:** In the cited literature, measurements and evaluation of the results are mostly not done systematically. To get a better understanding of measurement results, cross-influences have to be evaluated and critically assessed. The lack thereof might also be a reason for contradictory data. Furthermore, the test setup boundary conditions such as type of heat source, type of HDH-circle and more need to be evaluated, when comparing results.

Our intention is to overcome these shortcomings, using a modified test setup that allows for accurate characterisations of the influences of water temperature, liquid height, superficial air velocity and sieve plate orifice diameter on the humidification of air in a BCH. In combination with optical measurement methods, we intend to provide a deeper understanding of humidification of air in a broad and relevant measurement range. We state, that this understanding will help to design and construct BCHs in a more efficient manner; thus, finally also improving the efficiency of HDH-systems.

## 2 Methods and materials

### 2.1 Experimental setup

**Fig. 1:**
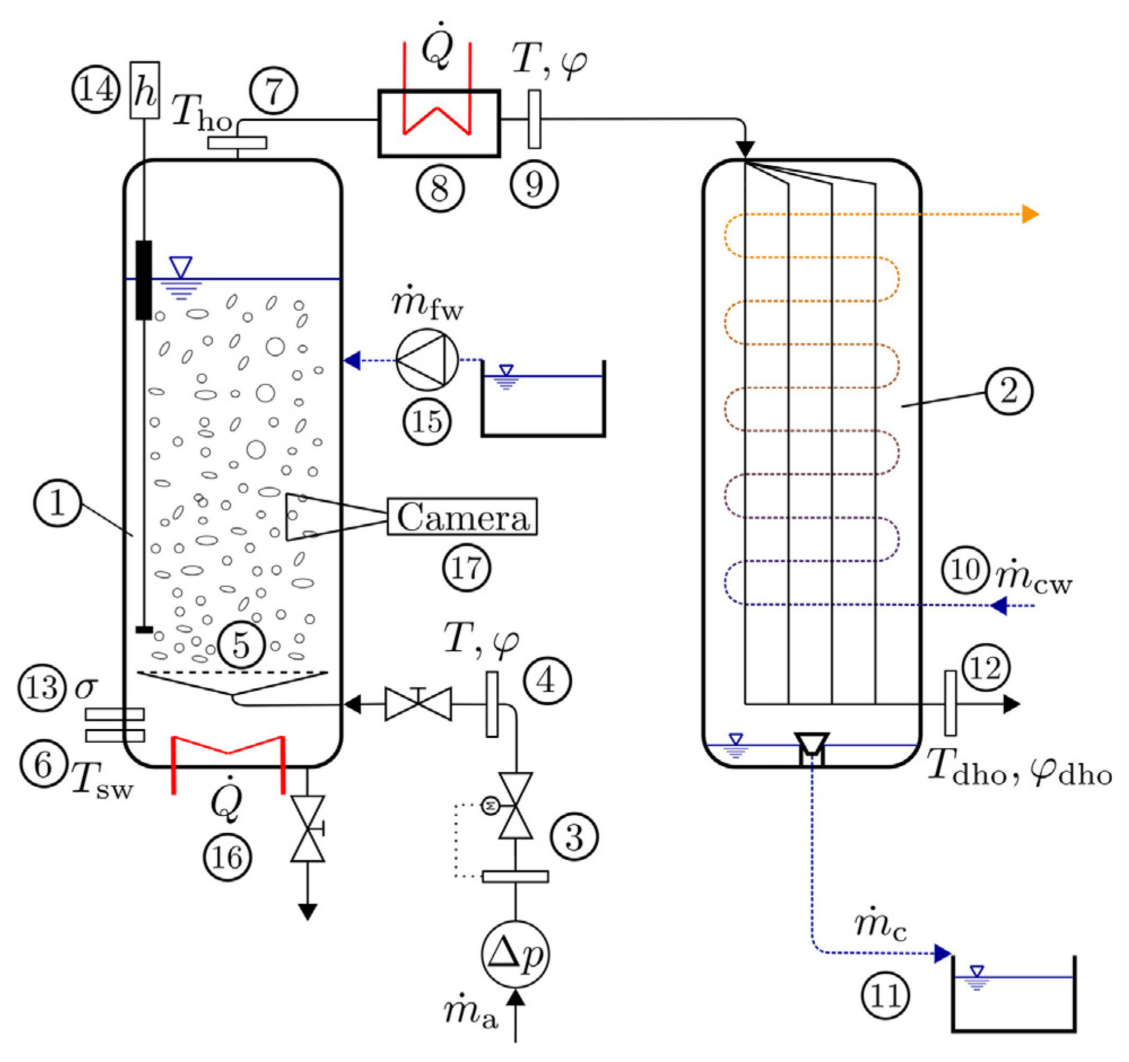
Experimental setup: (1) humidifier, (2) dehumidifier, (3) flow meter, (4,9,12) temperature-humidity sensors, (5) sparger plate, (6,7) resistance thermometers, (8) heating coil, (10) cooling water flow, (11) condensate flow, (13) electrical conductivity sensor, (14) liquid height sensor, (15) dosage pump, (16) heating cartridges and (17) compact system camera.

The main components of the setup shown in Fig. 1 are the BCH (1) for humidification and the shell-and-tube heat exchanger (2) for condensation.

A cylindrical BCH with optical access has been built out of stainless steel and acrylic glass parts. The cylindrical humidifier has a diameter of 140 mm. Its height can be varied by using different acrylic glass cylinders.

**Fig. 2:**
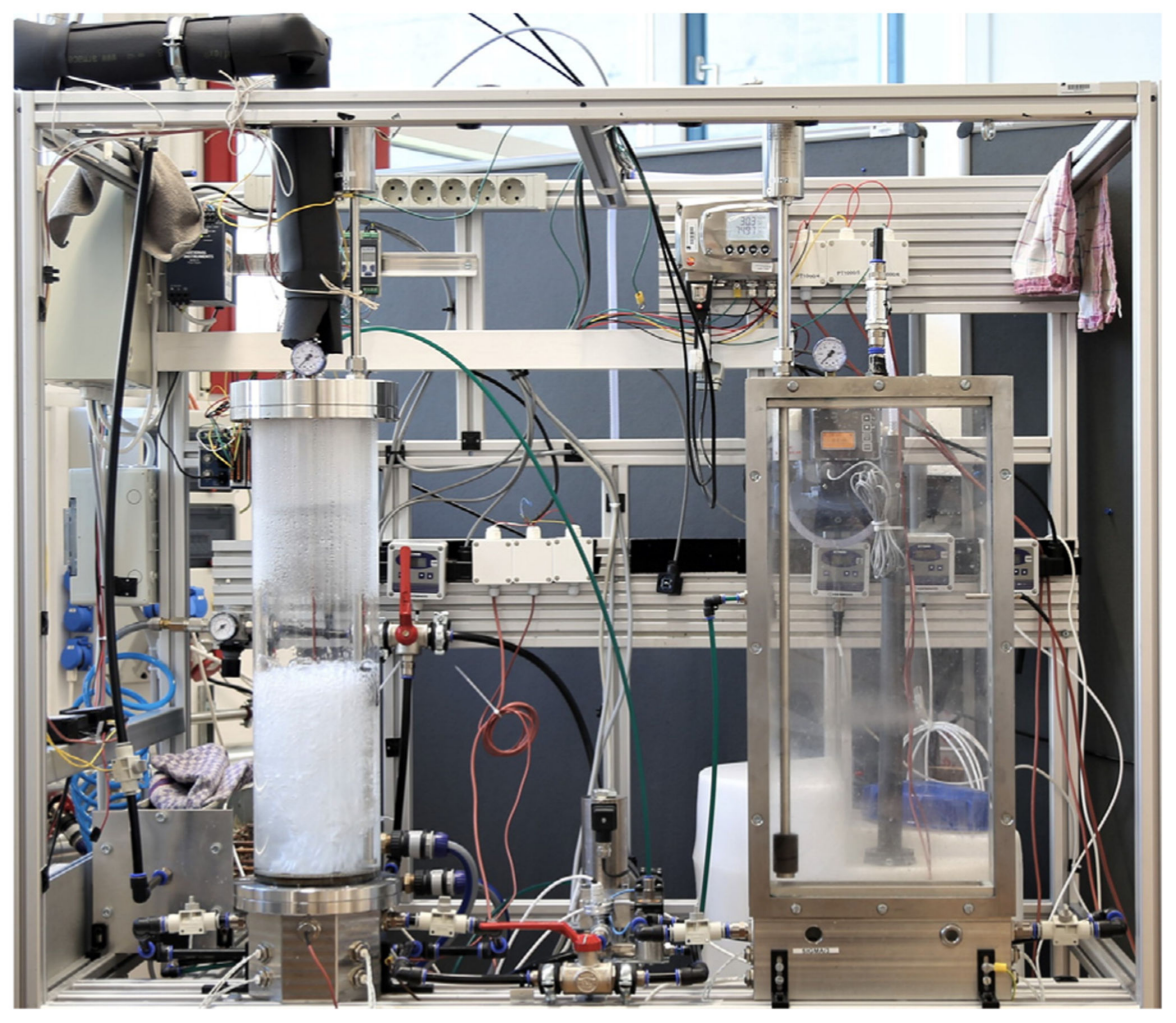
Experimental bubble column setup (Left hand side shows the cylindrical bubble column humidifier used for measurements).

The air mass flow into the humidifier is controlled using a flow meter (3), temperature and relative humidity of the inlet air stream are measured by a temperature-humidity sensor (4). The air stream enters the humidifier through a perforated sieve plate (5). By using different sieve plate geometries and by varying superficial air velocity, bubble characteristics can be varied. To measure water temperature in the humidifier (6) as well as air temperature at the outlet of the humidifier (7), resistance thermometers are installed. The hot and humid air stream at the humidifier outlet is further heated by a heating coil (8) to lower relative humidity of the stream, while maintaining a constant humidity ratio. This avoids condensation on the temperature-humidity sensor (9). Temperature and relative humidity after the heating coil (9) are measured by a temperature-humidity sensor for high temperatures and high humidities. Relative humidity at the humidifier outlet is calculated using the resistance thermometer (7) and humidity sensor (9) values. Subsequently, the humid air stream enters the dehumidifier, where the air is cooled by a cooling water flow (10) and the fresh water is collected as condensate (11). At the outlet of the dehumidifier, temperature and relative humidity of the air stream are measured once more using a temperature-humidity sensor (12), before the air is released into the ambient. To maintain constant values for salinity and liquid height in the BCH, a liquid height sensor (14) and a dosage pump (15) are installed. The dosage pump (15) adds fresh water to the humidifier to maintain a constant liquid height. As the amount of salt in the humidifier is constant, the salinity also stays the same. The liquid in the bubble column is heated by electric heating cartridges (16). A compact camera system (17) is used, to take images of the bubbly flow. The measurement instruments used in our HDH-setup are listed in Table 2 including their respective measurement ranges and accuracies. In Table 2, the number in the column Instrument indicates the sensor position in the setup as shown in Fig. 1. An image of the experimental setup is displayed in Fig. 2.

### 2.2 Measurement procedure

Following steps are taken for a typical measurement run:

- The system is powered and the cooling water line is opened.
- Sodium chloride is added to tap water to a weight percentage of 35g_sa_/kg_tw_, to simulate seawater. The BCH is manually filled to the designated liquid height.
- The air line is opened and superficial air velocity is set to the de signated value.
- Water and heating coil temperatures are set to the designated values.
- The measurement starts after the air temperature at the humidifier outlet reaches a steady-state value. Steady-state is defined by a temperature change of less than 0.5K in 30 min.
- The amount of condensate collected is measured in steady-state for 60 min.
- Images of the bubbly flow in the humidifier are taken for analysing flow characteristics.
- The maximum obtainable amount of condensate is calculated using thermodynamic laws and compared to the amount of condensate measured.

An acrylic glass cylinder with a height of 600 mm is used for the measurements. In general, no thermal insulation is applied to the humidifier, with the exception of the impact assessment of the liquid height in Section 3.3. Measurement parameters are varied in the ranges shown in Table 3. In order to evaluate the impact of certain measurement parameters, Eqs. (1)–(3) are used. The density of air at the humidifier inlet is assumed to be 1.2kg/m^3^. Superficial air velocity is calculated as the quotient of volumetric air flow rate ${\dot{V}}_{a}$ and cross-section of the cylindrical BCH *A_cs_*:

(1) $$v_{\text{as}}=\frac{{\dot{V}}_{a}}{A_{\text{cs}}}$$

The amount of condensate ${\dot{m}}_{c}$ can be calculated using air mass flow *ṁ_a_*, humidity ratio of the humidifier outlet *ω*_ho_ and humidity ratio of the dehumidifier outlet *ω*_dho_.

(2) $${\dot{m}}_{\text{c}}={\dot{m}}_{\text{a}}\cdot \left(\omega_{\text{ho}}-\omega_{\text{dho}}\right)$$

For evaluating humidity ratio of air, Eq. (3) is used, where φ is relative humidity, *p*_s_ is water vapor saturation pressure and *p*_atm_ is atmospheric pressure of the air. The water vapor saturation pressure *p*_s_ is evaluated using the renowned Antoine equation.

(3) $$\omega =0.622.\frac{\phi \cdot p_{\text{s}}}{p_{\text{atm}}-\phi \cdot p_{s}}$$

After the evaluation of several preliminary test runs with various boundary conditions and parametric settings, the following assumptions can be made:

- Temperature and salinity profile of the water in the humidifier is assumed to be uniform; pressure in the BCH is assumed to be the atmospheric pressure of 1 atm.
- For calculations of the maximum obtainable amount of condensate $\left({\dot{m}}_{\text{c},\max}\right),$ the humidity ratio between humidifier and dehumidifier outlet is calculated and multiplied with the air mass flow using Eqs. (1)–(3).
  – Air at the humidifier outlet is assumed to be saturated and at bulk water temperature.
  – Air at the dehumidifier outlet is assumed to be saturated and at a temperature of 12.8 °C.
- We define a theoretical productivity of a measurement run as the amount of condensate, that would be produced, if at both humidifier and dehumidifier outlet the air temperatures of a reference state were reached instead of the actual air temperatures. For calculating such a theoretical amount of condensate ${\dot{m}}_{\text{c},\text{theo}}$ using Eqs. (1)–(3), our assumptions are:
  – Air is saturated at both humidifier and dehumidifier outlet.
  – The highest measured temperature at the humidifier outlet (7) is used as a reference value for the outlet temperature.
  – The corresponding temperature value of the reference measurement is used as a temperature reference value for the dehumidifier outlet (12).

To calculate this theoretical amount of condensate produced with eliminated temperature differences, humidity ratio differences are calculated between the measurement and a reference state for both the humidifier and dehumidifier outlet temperatures (Eqs. (4) and (5)).

(4) $$\text{Δ}\omega_{\text{ho}}=\omega_{\text{ho,m}}-\omega_{\text{ho,ref}}$$

(5) $$\text{Δ}\omega_{\text{dho}}=\omega_{\text{dho,m}}-\omega_{\text{dho,ref}}$$

Using Eq. (2) in combination with Eqs. (4) and (5), condensate amount differences are calculated for both humidifier and dehumidifier outlet. These condensate amount differences are subtracted from the measured amount of condensate, to calculate the theoretical amount of condensate produced (Eq. (6)).

(6) $${\dot{m}}_{\text{c,theo}}={\dot{m}}_{\text{c}}-\text{Δ}{\dot{m}}_{\text{c,ho}}-\text{Δ}{\dot{m}}_{\text{c,dho}}$$

## 3 Results and discussion

In this section, our experimental setup is validated using the impact assessment of water temperature and our results regarding the influences of superficial air velocity, liquid height and sieve plate orifice diameter are shown and critically evaluated.

### 3.1 System validation

**Fig. 3:**
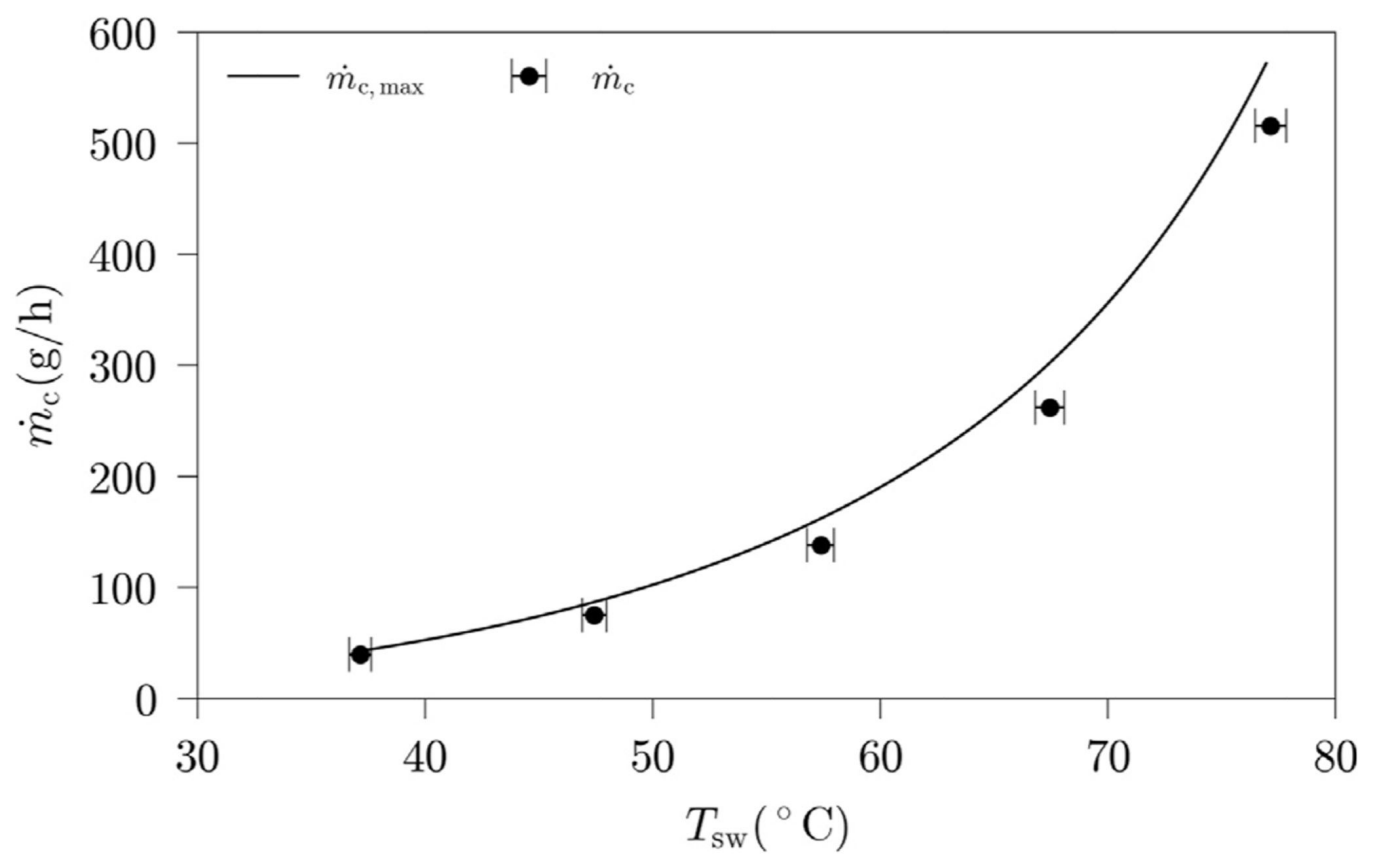
HDH-system productivity in dependence of water temperature (Benchmark settings: *h =* 200 mm, *v*_as_ = 2 cm/s, *d*_sp_ = 3 mm; error bars indicate the sensor accuracy of the measurements).

To validate our setup, the impact of water temperature on the system productivity is characterised. This can be done, as this impact is well discussed in literature. Fig. 3 shows the amount of condensate produced for different water temperatures in the humidifier as well as the maximum obtainable amount of condensate for the used parameter settings.

The productivity of the HDH-system increases with an increase in water temperature. As expected, the productivity increase with water temperature is exponential. The achieved productivities are slightly below the maximum obtainable condensate amount. This can be either due to an insufficient humidification or to heat loss over the humidifier surface. However, Fig. 3 clearly indicates, that our HDH-setup including the measurement devices and methods is validated and suited for systematic studies of the humidification process.

### 3.2 Impact of superficial air velocity

**Fig. 4:**
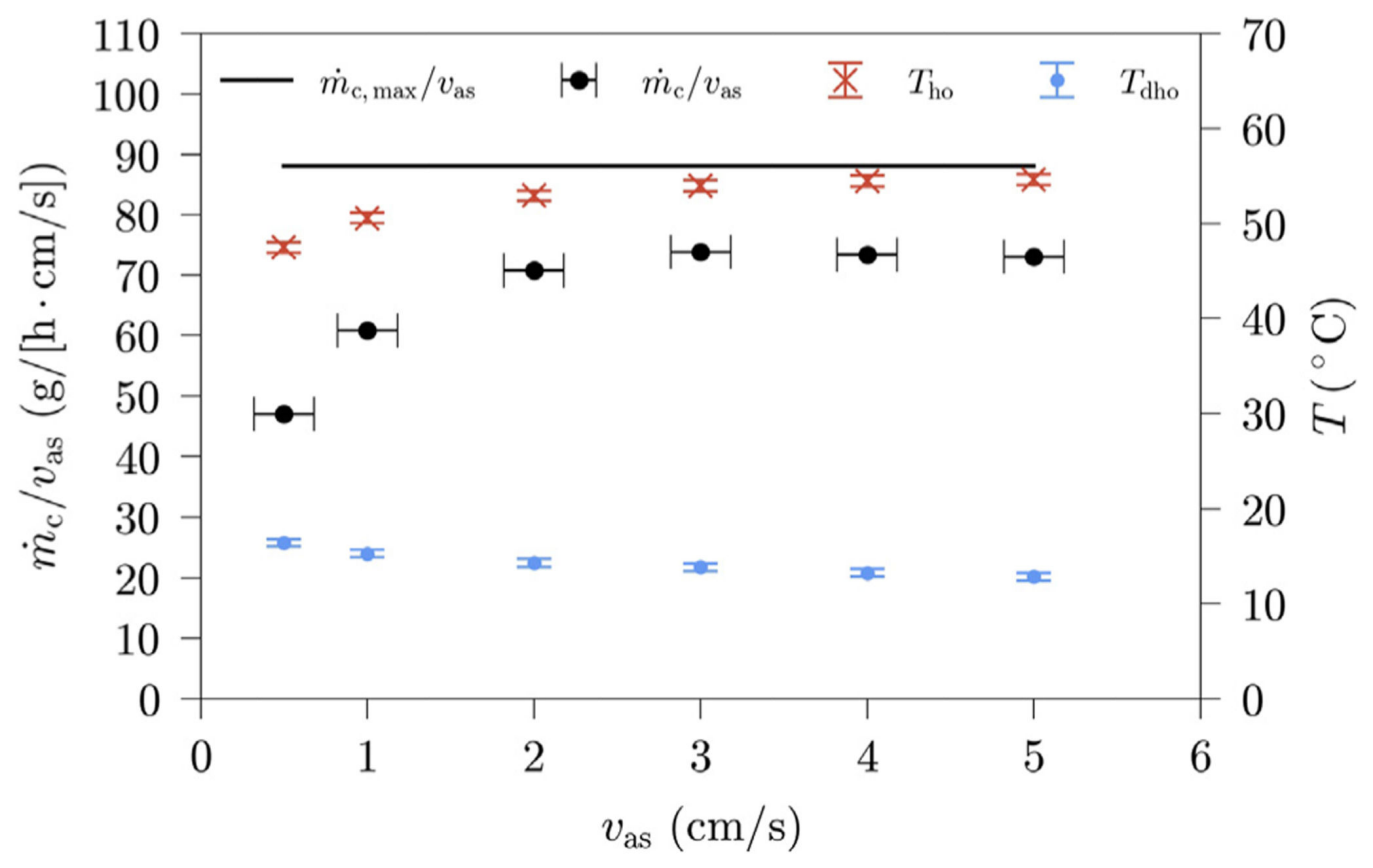
HDH-system productivity and selected air temperatures in dependence of superficial air velocity (Benchmark settings: *T*_sw_ = 60 °C, *h* = 200 mm, *d*_sp_ = 3 mm; error bars indicate the sensor accuracy of the measurements).

The dependence of the HDH-system productivity on superficial air velocity is displayed in Fig. 4. The productivity shown in Fig. 4 is normalized to a superficial air velocity of 1 cm/s, to provide a better comparability. The absolute system productivity even increases with an increase in superficial air velocity, if the humidification is unaffected by it, as more air with the same humidity ratio, thus more water vapor leaves the humidifier and can subsequently be condensed.

However, Fig. 4 indicates, that superficial air velocity does impact the humidification, as the normalized system productivity is affected by it. The normalized productivity increases with increasing superficial air velocity, gradually reaching a limit. This increase might be due to temperature changes at the humidifier and the dehumidifier outlet. An increase in superficial air velocity leads to an increase of air tempera-ture at the humidifier outlet and a decrease at the dehumidifier outlet, also gradually reaching a limit. To investigate this hypothesis further, we calculate theoretical amounts of condensate $\left({\dot{m}}_{\text{c},\text{theo}}\right)$ for each mea-surement. The measurement run with a superficial air velocity of 7 cm/s is chosen as reference state, since the highest humidifier outlet temperature has been reached for this measurement.

**Fig. 5:**
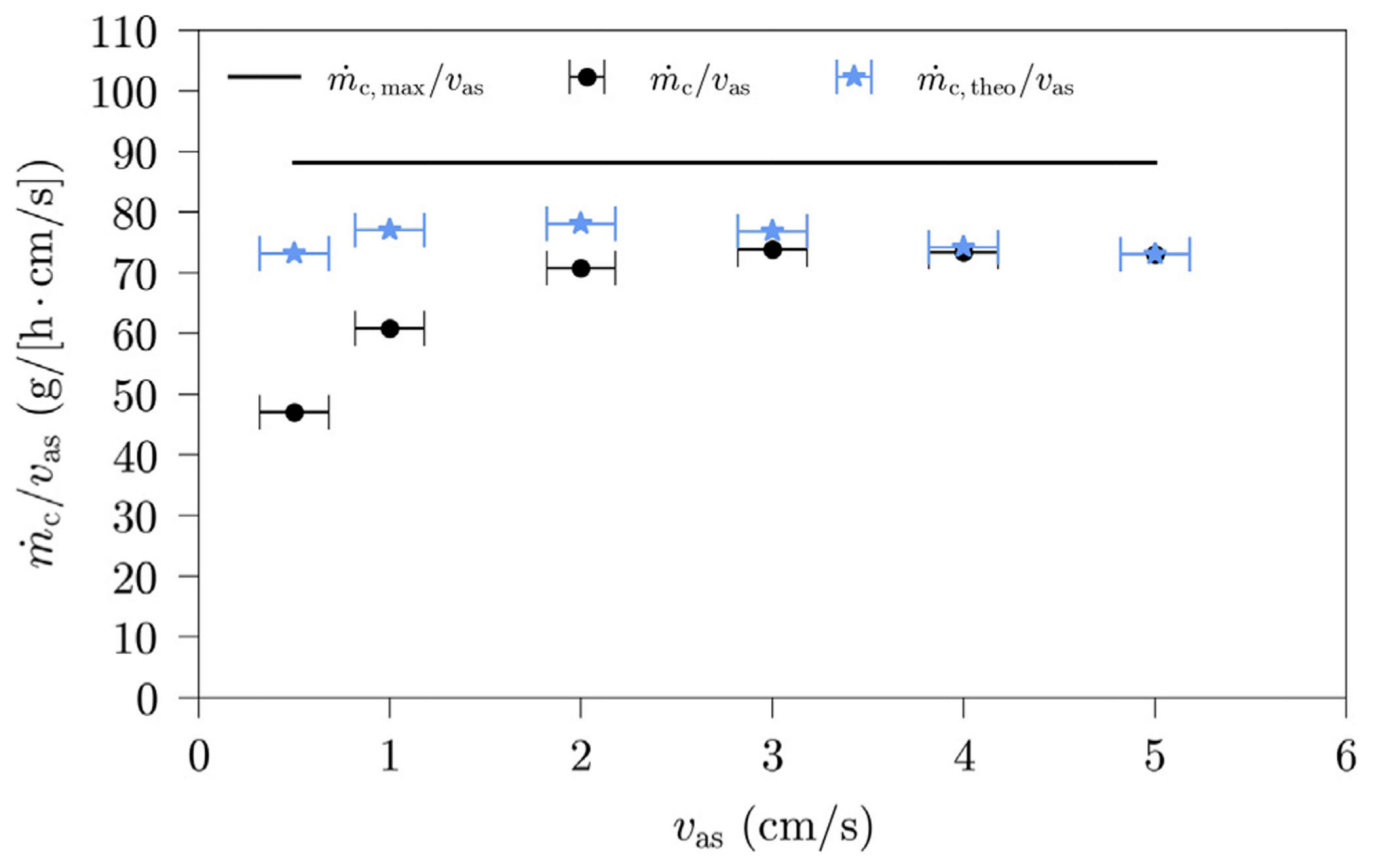
Comparison between measured and theoretical HDH-system productivity in dependence of superficial air velocity (Benchmark settings: *T*_sw_ = 60 °C, *h* = 200 mm, *d*_sp_ = 3 mm; error bars indicate the sensor accuracy of the measurements).

For further analysis, in Fig. 5, the normalized productivity is compared with the calculated theoretical productivity. The measurement data and the results of these calculations are shown in Table 4. Both Fig. 5 and Table 4 indicate that the normalized condensate difference is caused by the temperature differences in the humidifier, respectively the dehumidifier. Using the results of Table 4, it is estimated, that the temperature differences in the humidifier, respectively the dehumidifier, are responsible for an average of 89% and 11% of the change in normalized condensate production.

**Fig. 6:**
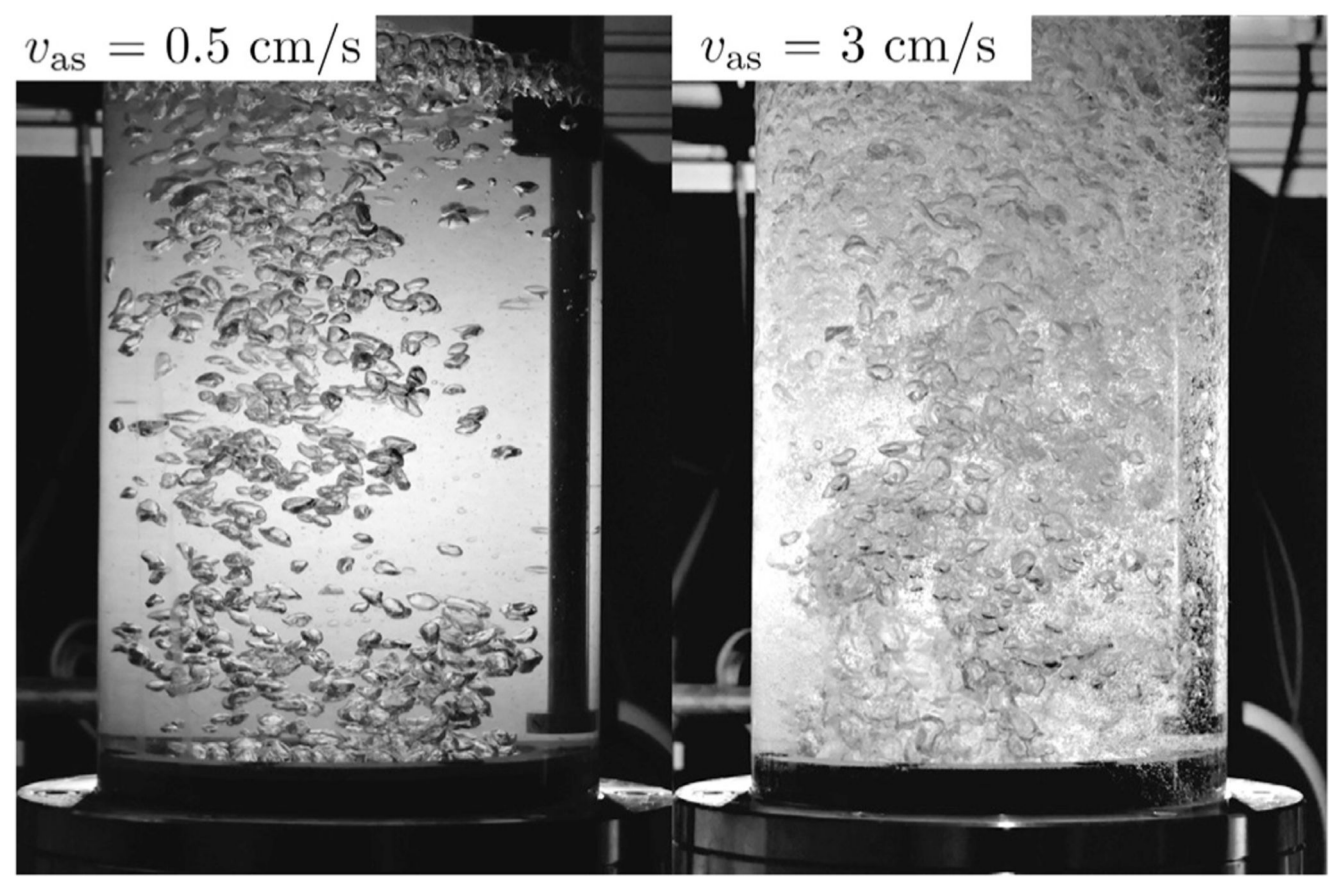
Bubble Column Images for two different superficial air velocities (0.5 and 3 cm/s), corresponding to a homogeneous (laminar character) and heterogeneous (turbulent character) flow regime respectively.

In Fig. 6, the bubble column flow regime is visualized for superficial air velocities of 0.5 and 3 cm/s, respectively. It can be seen, that a superficial air velocity of 0.5cm/s corresponds to a homogeneous flow regime. A clear indicator of this flow regime are homogeneous bubble sizes. A superficial air velocity of 3 cm/s, on the other hand, corresponds to a more heterogeneous flow. This flow regime is dominated by bubble coalescence and therefore leads to bubble conglomerates and consequently more turbulence. The heterogeneous flow regime also leads to the production of tiny spherical bubbles, which are visible at superficial air velocities greater than 3 cm/s, as they make the bubble regime more blurry. These tiny spherical bubbles are only forming for seawater, they have not been forming in preliminary measurements with fresh water. Our results of Figs. 4 and 5 can be linked with these flow regimes. It is concluded, that the normalized system productivity is strongly affected by the superficial air velocity for a homogeneous flow regime, with a decreasing influence, the more heterogeneous, the flow regime gets. Eventually, once a certain turbulence and heterogeneity is reached, the normalized system productivity is no longer affected by the superficial air velocity. Our impact characterisation agrees with the impact assessments of the majority of the previous studies [8,17–19], with the exception of an experimental study conducted by Liu and Sharqawy [24].

### 3.3 Impact of liquid height

**Fig. 7:**
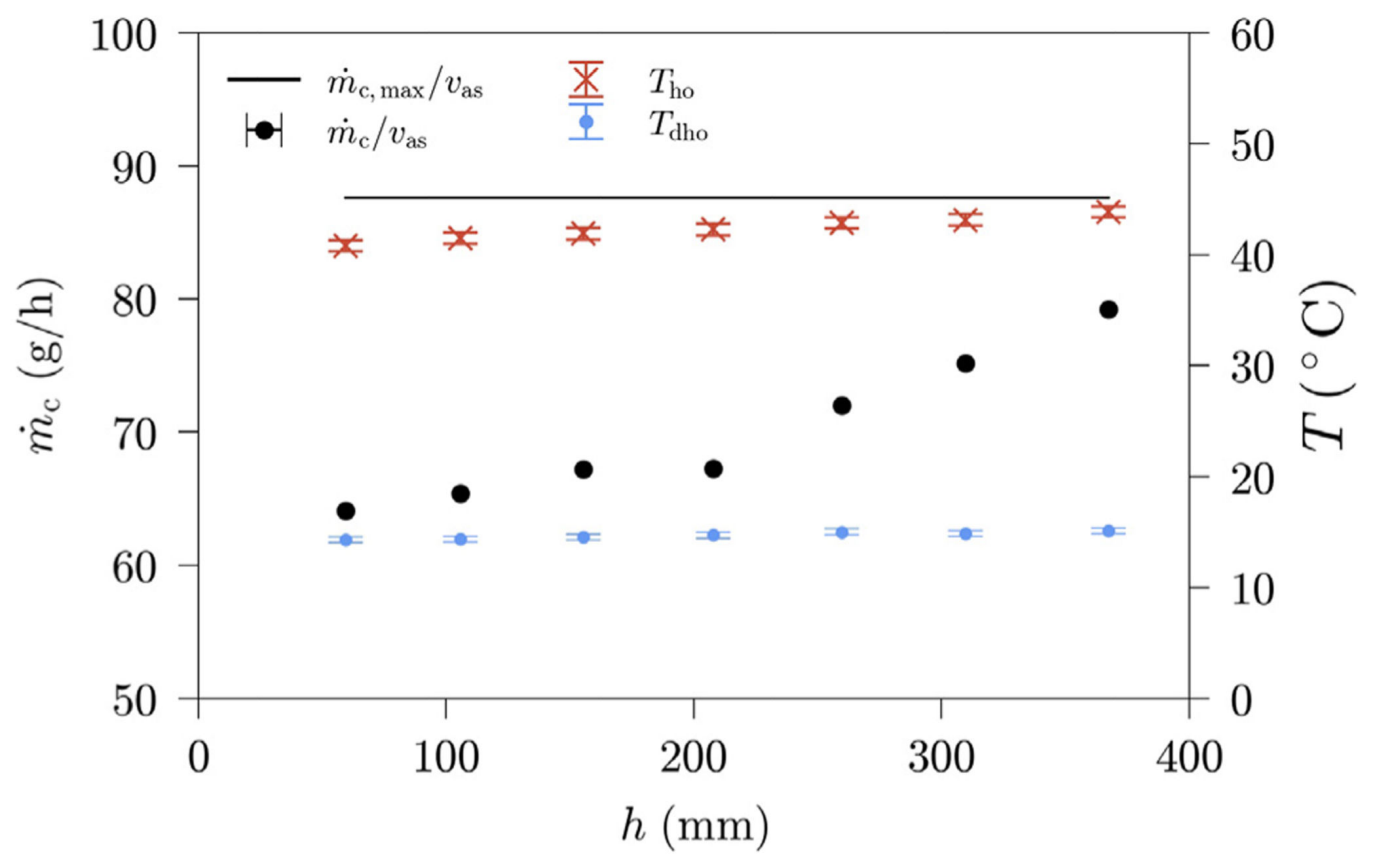
HDH-system productivity and selected air temperatures in dependence of liquid height; Benchmark settings: *T*_sw_= 50 °C, *v*_as_ = 2 cm/s, *d*_sp_ = 3 mm.

Fig. 7 shows the amount of condensate produced for varying liquid heights. Moreover, the temperatures at the outlets of the humidifier and the dehumidifier, respectively, have been plotted for each measurement. Since the free surface is fluctuating in operation, a mean value of the aerated height is calculated for every measurement.

An increase in system productivity with increasing liquid height is evident over the whole measurement range. This increase is caused by a simultaneous increase in air stream temperature at the humidifier outlet. The dehumidifier outlet temperature is affected only slightly by the changes in liquid height, the highest and the lowest measured temperatures at the dehumidifier outlet are only 0.98K apart.

**Fig. 8:**
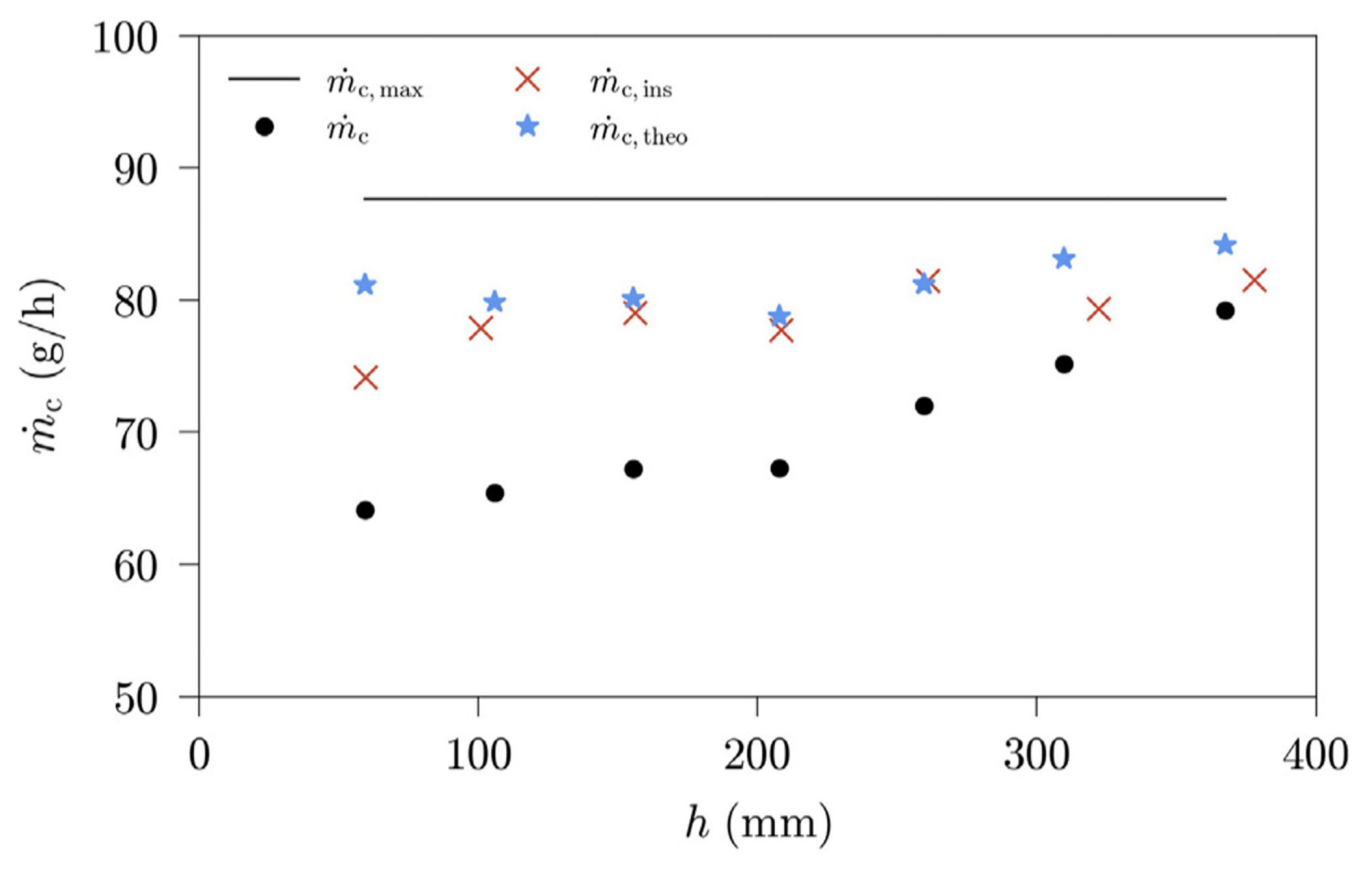
Measured and calculated HDH-system productivities in dependence of liquid height; Benchmark settings: *T*_sw_ = 50 °C, *v*_as_ = 2 cm/s, *d*_sp_ = 3 mm.

The increase in productivity with increasing liquid height can have two reasons: (1) it can be due to an enhanced heat and mass transfer in the BCH, or (2) it can be due to a smaller heat loss surface with increasing liquid height in the BCH. We define the surface of the humidifier, that is not covered by water, as the heat loss surface. To evaluate this, another measurement series is conducted, using a thermally insulated BCH. Additionally, a theoretical productivity is calculated once more for every measurement conducted. Since the measurements will be compared with a second measurement series where the bubble column is thermally insulated, the highest humidifier outlet temperature in the thermally insulated case is chosen as reference temperature value. This measurement corresponds to a humidifier outlet temperature of 44.69 °C and a dehumidifier outlet temperature of 14.15 °C. The measurement data including the reference measurement and the theoretical productivity are shown in Table 5. The two measurement runs and the theoretical productivity are visualized in Fig. 8, for comparative purposes.

Fig. 8 indicates, that the increase in productivity is caused by a smaller heat loss surface with increasing liquid height. Both the measurement series for the thermally insulated case and the theoretical productivity lead to very similar condensate productions, showing a negligible influence of the liquid height on the HDH-system productivity. This impact characterisation agrees well with previous works by El-Agouz as well as Liu and Sharqawy [8,24]. Two more previous works noticed a positive impact of liquid height on the productivity [17,18]. However, since the impact in those works is very small, the changes in productivity can be explained by heat loss phenomena, as it is the case for this work. Finally, the liquid height had a negative influence on the humidification in an experimental work by Abd-Ur-Rehman [19]. In their experimental setup, however, the bubble column temperature was not maintained constant, since only the inlet water was heated.

For the measurements conducted in this study, the maximum ob-tainable productivity is not achieved for any measurement, but a clear improvement with thermal insulation is evident. Hence, it can be concluded, that for a thermally well insulated BCH design, the liquid height difference does not impact the humidification of the air for the studied measurement range. Our calculations show, that the humidifier and dehumidifier outlet temperature differences are responsible for an estimated 95.5% and 4.5% of the measured differences in condensate production.

### 3.4 Impact of sieve plate orifice diameter

**Fig. 9:**
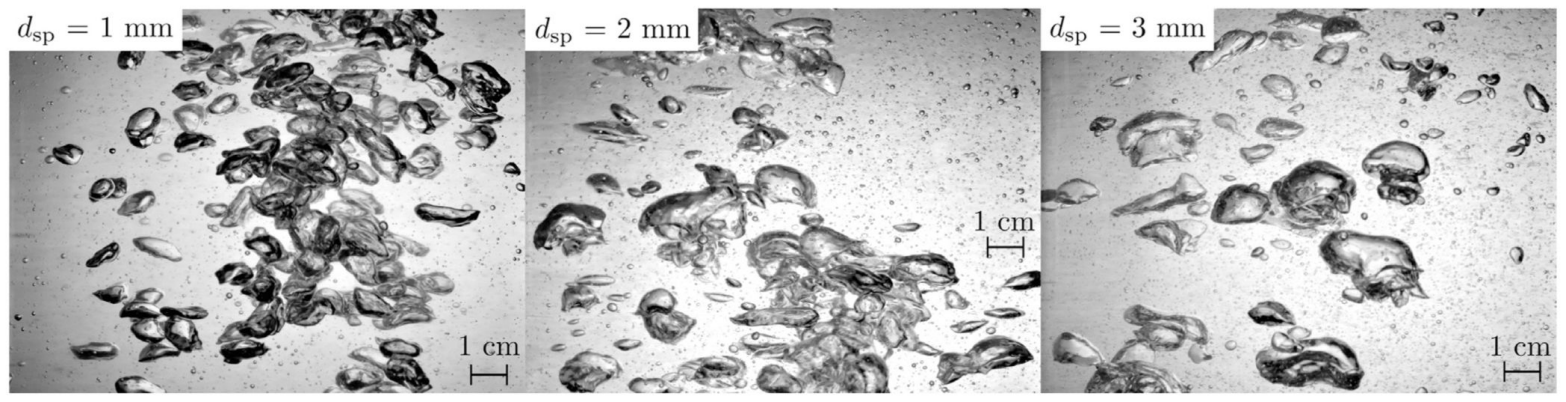
Bubble column flow regime for different sieve plate orifice diameters.

Fig. 9 shows the resulting bubble column flow regimes for the three tested sieve plate orifice diameters. It is evident, that with an increase in sieve plate orifice diameter, the mean bubble size also increases. This fits the description of Kantarci et al. [26], that sparger design greatly impacts the resulting bubble size, and that smaller orifice diameters result in smaller bubble sizes. Furthermore, Fig. 9 indicates, that a larger sieve plate orifice diameter results in bubble coalescences and in the formation of bigger bubbles.

**Fig. 10:**
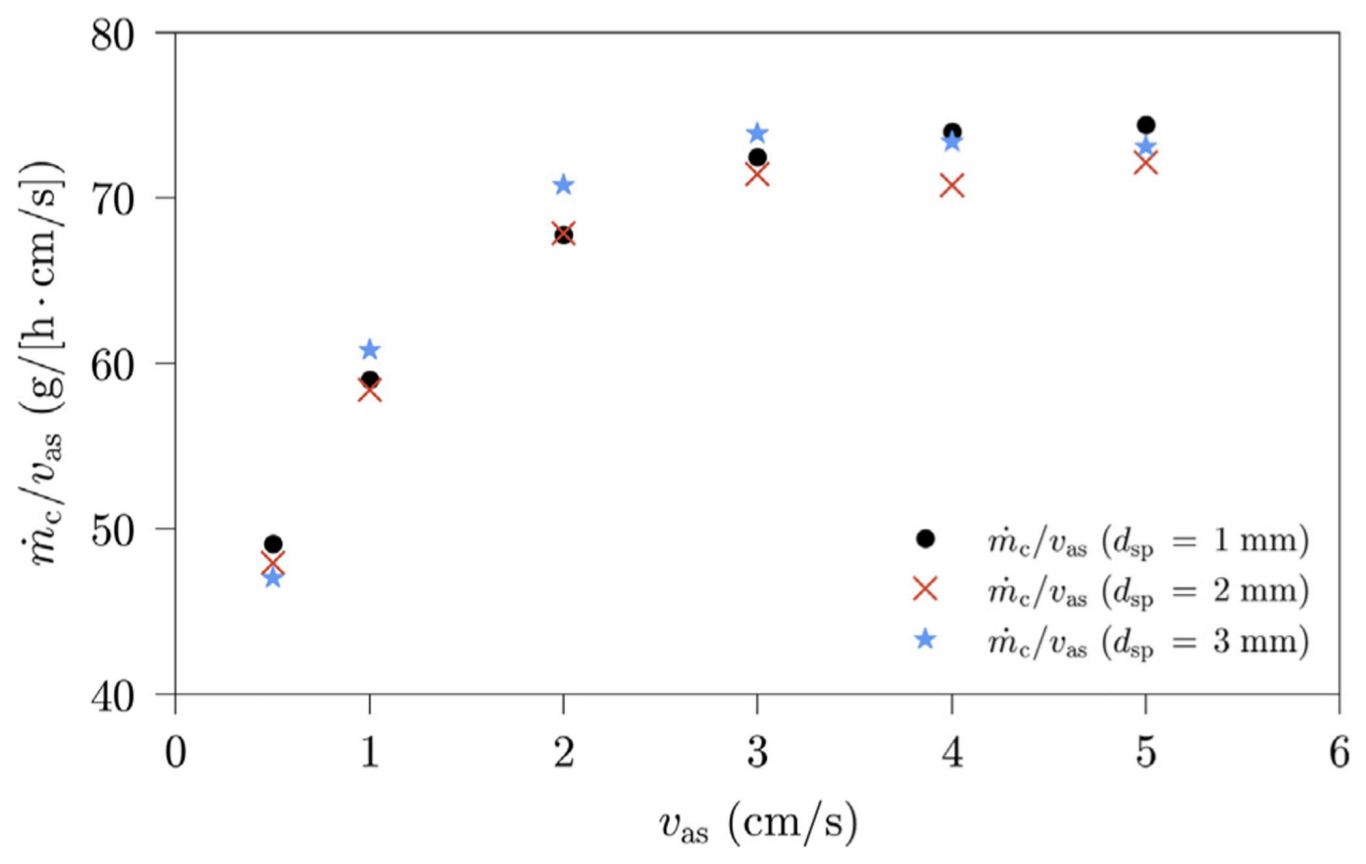
HDH-system productivity in dependence of superficial air velocity for different sieve plate orifice diameters; Benchmark settings: *T*_sw_ = 50 °C, *h* = 200 mm, *d*_sp_ = 3 mm.

The influence of sieve plate orifice diameter on the amount of condensate produced is displayed in Fig. 10. Measurement runs are conducted for three different sieve plate orifice diameters with a variation of the superficial air velocity. Our results show, that the normalized productivity of the HDH-system is not considerably influenced by sieve plate orifice diameter for the diameters investigated.

For a more detailed assessment of the impact of flow characteristics and especially bubble size, further measurements and analyses, for different sieve plate geometries and measurement ranges, are necessary.

## 4 Conclusion

A new test setup for the humidification of air in bubble columns is presented. With it, experimental investigations of the humidification gain a more systematic character. Optical access to the bubble column is crucial and allows to add evaluations of the flow characteristics to the experimental data.

Our experimental study shows, that by (1) comparing our measurement data with an optical assessment of the flow, (2) choosing a broad and relevant measurement range for every parameter and (3) cross-verifying and evaluating the measurement data, a better understanding of the humidification in bubble columns can be gained.

Our test setup and these results shall serve as a base in getting an even deeper understanding of the humidification process of air in bubble columns. It is planned, to collect more experimental data, to subsequently obtain semi-empirical correlations that describe the humidification of air in seawater as a function of the parameters impacting it. These correlations are important, to improve thermodynamic models and design recommendations for bubble column based HDH-systems. Ultimately, our work aims to provide a relevant contribution to improving the design and efficiency of HDH-systems.

## Figures and Tables

**Table 1:** Measurement ranges tested and impact of operational parameters on humidi-fication/HDH-system productivity (+ + strong positive impact; + positive impact; ~ no impact; − negative impact).

Article	*T_sw_* (°C)	Impact *T_sw_*	*ṁ_a_* (kg/h)	Impact *ṁ_a_*	*h* (mm)	Impact *h*
[8,21]	50–87	+ +	4.2–14	+	200–600	~
[17]	48 − 75	+ +	11–17.7	+	50–200	+
[18]	30–75	+ +	48–143	+	17–70	+
[19]	35–75	+ +	61.1–91.6	+	10–50	−
[20]	n/a	n/a	50.4–64.8	+	80–120	−
[22]	n/a	n/a	50.4–64.8	+	n/a	n/a
[23]	n/a	n/a	45.8–91.6	+	10–50	~
[24]	n/a	n/a	1–4.6	−	50–70	~
[25]	n/a	n/a	0.3–0.6	− and +	50–100	− and +

**Table 2:** Measurements instruments, measurement ranges and accuracies.

Parameter	Instrument	Measurement Range	Measurement Accuracy
Temperature	PT1000 thermometer (4,6,7,12)	0–100 °C	± 0.3 + 0.005·*T* (°C)
Liquid height	Magnetostrictive level (14)	0–500 mm	± 0.5 (mm)
Relative humidity	Capacitive polymer sensor (4,12)	0–100 %RH	± 2.5 (%RH)
Temperature	PT1000 thermometer (9)	0–180 °C	± 0.1 + 0.0017·*T* (°C)
Relative humidity	Capacitive polymer sensor (9)	0–100 %RH	± 1 + 0.007·*φ* (%RH)
Electrical conductivity	Capacitive polymer sensor (13)	0–500 mS/cm	± 2.5 + 0.005·*σ* (mS/cm)
Volumetric air flow rate	Flow meter (3)	0–10 m^3^/h	± 0.1 (m^3^/h)
Condensate amount	Scale	0–500 g	± 0.01 (g)

**Table 3:** Parameter variation ranges.

Parameter	Variation range
Water temperature	40–80 °C
Superficial air velocity	0.5–5 cm/s
Liquid Height	60–378 mm
Sieve plate orifice diameter	1–3 mm

**Table 4:** Impact estimation for superficial air velocity.

Measurement	*v*_as_ (cm/s)	${\dot{m}}_{\text{c}}/v_{\text{as}}\;\left(\text{g}/\left[{\text{h}}^{\text{.}}\;\text{cm/s}\right]\right)$	*T*_ho_ (°C)	*T*_dho_ (°C)	${\dot{m}}_{\text{c}}/v_{\text{as}}\;\left(\text{g}/\left[{\text{h}}^{\text{.}}\;\text{cm/s}\right]\right)$	$\text{Δ}{\dot{m}}_{\text{c,dho}}/v_{\text{as}}\;\left(\text{g}/\left[{\text{h}}^{\text{.}}\;\text{cm/s}\right]\right)$	${\dot{m}}_{\text{c,theo}}/v_{\text{as}}\;\left(\text{g}/\left[{\text{h}}^{\text{.}}\;\text{cm/s}\right]\right)$
1	0.5	47.00	47.50	16.41	−24.60	−1.63	73.23
2	1.0	60.80	50.57	15.26	−15.19	−1.07	77.07
3	2.0	70.78	52.94	14.30	−6.73	−0.63	78.14
4	3.0	73.86	54.00	13.84	−2.56	−0.43	76.86
5	4.0	73.38	54.46	13.23	−0.67	−0.17	74.22
6/Reference	5.0	73.07	54.62	12.81	0.00	0.00	73.07

**Table 5:** Impact estimation for liquid height.

Measurement	*h*_mean_ (mm)	${\dot{m}}_{c}\;\left(\text{g}/h\right)$	*T*_ho_ (°C)	*T*_dho_(°C)	$\text{Δ}{\dot{m}}_{\text{c,ho}}\;\left(\text{g}/h\right)$	$\text{Δ}{\dot{m}}_{\text{c,dho}}\;\left(\text{g}/h\right)$	${\dot{m}}_{\text{c,theo}}\;\left(\text{g}/h\right)$
1	60	63.34	40.80	14.33	−16.86	− 0.16	81.11
2	106	64.09	41.47	14.38	−14.20	− 0.20	79.83
3	155	65.42	41.88	14.55	−12.53	− 0.36	80.09
4	208	67.20	42.26	14.72	−10.95	− 0.51	78.72
5	260	71.99	42.85	15.02	− 8.42	− 0.79	81.20
6	301	75.18	43.11	14.89	− 7.28	− 0.67	83.13
7	367	79.19	43.83	15.13	− 4.04	− 0.89	84.12
Reference	378	81.51	44.69	14.15	0.00	0.00	81.51

## Nomenclature

**Acronyms**
BCH: Bubble column humidifier
HDH: Humidification-dehumidification

**Physical properties**
*ṁ*: Mass flow (kg/s)
*$\dot{Q}$*: Heat flow (W)
$\dot{V}$: Volumetric air flow rate (m^3^/h)
*ω*: Humidity ratio of air (g_v_/kg_a_)
*σ*: Electrical conductivity (mS/cm)
*φ*: Relative humidity of air (%RH)
*A*: Area (m^2^)
*h*: Liquid height (m)
*p*: Pressure (atm)
*T*: Temperature (°C)
*v*: Velocity (cm/s)

**Subscripts**
as: Air superficial
atm: Atmospheric
a: Air
b: Brine
cs: Cross-section
cw: Cooling water
c: Condensate
dho: Dehumidifier outlet
est: Estimated
fw: Fresh water
hc: Heating coil
ho: Humidifier outlet
ins: Thermally insulated
max: Maximum
mean: Mean value
m: Measurement
ref: Reference measurement
sa: Salt
sp: Sieve plate
sw: Seawater
s: Saturated
theo: Theoretical value
tw: Tap water
v: Water vapor
